# Smoking increases rectal cancer risk to the same extent in women as in men: results from a Norwegian cohort study

**DOI:** 10.1186/1471-2407-14-321

**Published:** 2014-05-06

**Authors:** Ranjan Parajuli, Eivind Bjerkaas, Aage Tverdal, Loïc Le Marchand, Elisabete Weiderpass, Inger T Gram

**Affiliations:** 1 Department of Community Medicine, Faculty of Health Sciences, UiT, The Arctic University of Tromsø, Tromsø, Norway; 2 Division of Epidemiology, Department of Pharmacoepidemiology, Norwegian Institute of Public Health, P.O. Box 4404, Nydalen, 0403 Oslo, Norway; 3 Epidemiology Program, University of Hawaii Cancer Center, Honolulu, HI, USA; 4 Department of Medical Epidemiology and Biostatistics, Karolinska Institutet, Stockholm, Sweden; 5 Department of Genetic Epidemiology, Samfundet Folkhälsan, Helsinki, Finland; 6 Department of Research, Cancer Registry of Norway, Oslo, Norway; 7 Norwegian Centre for Integrated Care and Telemedicine, University Hospital of North Norway, Tromsø, Norway

**Keywords:** CONOR, Cigarette smoking, Rectal cancer, Cohort, Norway

## Abstract

**Background:**

Smoking has recently been established as a risk factor for rectal cancer. We examined whether the smoking-related increase in rectal cancer differed by gender.

**Methods:**

We followed 602,242 participants (49% men), aged 19 to 67 years at enrollment from four Norwegian health surveys carried out between 1972 and 2003, by linkage to Norwegian national registries through December 2007. Hazard ratios (HRs) and 95% confidence intervals (CIs) were estimated by fitting Cox proportional hazard models and adjusting for relevant confounders. Heterogeneity by gender in the effect of smoking and risk of rectal cancer was tested with Wald *χ*^2^.

**Results:**

During a mean follow-up of 14 years, 1,336 men and 840 women developed invasive rectal cancer. Ever smokers had a significantly increased risk of rectal cancer of more than 25% for both men (HR = 1.27, 95% CI = 1.11-1.45) and women (HR = 1.28, 95% CI = 1.11-1.48) compared with gender-specific never smokers. Men smoking ≥20 pack-years had a significantly increased risk of rectal cancer of 35% (HR = 1.35, 95% CI = 1.14-1.58), whereas for women, it was 47% (HR = 1.47, 95% CI = 1.13-1.91) compared with gender-specific never smokers. For both men and women, we observed significant dose–response associations between the risk of rectal cancer for four variables [Age at smoking initiation in years (both p_trend_ <0.05), number of cigarettes smoked per day (both p_trend_ <0.0001), smoking duration in years (p_trend_ <0.05, <0.0001) and number of pack-years smoked (both p_trend_ <0.0001)]. The test for heterogeneity by gender was not significant between smoking status and the risk of rectal cancer (Wald *χ*^2^, p -value; current smokers = 0.85; former smokers = 0.87; ever smokers = 1.00).

**Conclusions:**

Smoking increases the risk of rectal cancer to the same extent in women as in men.

## Background

An expert group at the International Agency for Research on Cancer (IARC) recently re-evaluated the carcinogenetic effects of smoking in humans, and concluded that smoking is a risk factor for both colon and rectal cancer [1]. In a recently published study based on present cohort, we found that the increased risk of colon cancer due to cigarette smoking may be greater in women than men [2].

The variation in the smoking epidemic by country and gender was first described in a model focusing on the four stages of the tobacco epidemic in Western countries [3] and later in a more gender-specific model [4]. In Norway, the prevalence of daily smoking was around 25% for women and 65% for men in the 1950s. During the early 1970s, it increased to 32% for women and decreased to 52% for men. Since then, the prevalence of daily smoking has decreased steadily among men, while a decrease among women started only at the turn of the millennium. In 2007, about 24% of Norwegian men and women, aged 16 to 74 years were daily smokers [5,6].

During the last 50 years, the incidence rate of rectal cancer has increased dramatically in Norway. It was about 5 per 100,000 for women and 6 per 100,000 for men in the late 1950s. In 2007, which was the end of the follow-up period in our study, the risk had more than doubled to 12 per 100,000 for women and 17 per 100,000 for men [7].

The main purpose of our study was to examine if the smoking-related increase in rectal cancer differed by gender in a large Norwegian cohort.

## Methods

### Study population

The cohort included 652,792 Norwegians (49% men), most of whom were aged 19 to 67 years at enrollment, who participated in four different Norwegian health surveys initiated by the National Health Screening Service (now included in the Norwegian Institute of Public Health). These surveys were conducted between 1972 and 2003: the Oslo study I (1972–1973), the Norwegian counties study (1974–1988), the 40 years cohort (1985–1999) and the Cohort of Norway (CONOR, 1994–2003). The design and protocol of these surveys were very similar, but there were some modifications made during different time periods, mainly to the questionnaires, regarding smoking, alcohol consumption, physical activity and other lifestyle factors [8-13].

Information was gathered through a baseline questionnaire and a short health examination. In most surveys, the participants were given a supplementary questionnaire, which they completed at home and mailed back in a pre-stamped envelope. The participation rates for the different surveys varied from 56% to 88% [13]. The present study was approved by the Regional Committee for Medical Research Ethics South-East, Norway. More details about the study population are described elsewhere [2,14].

### Exposure information

The smoking questions across the four health surveys were similar, but not identical. All surveys had a baseline questionnaire, which included a detailed assessment of smoking habits, physical activity, and other lifestyle factors. The questionnaires included questions on current and former smoking habits, smoking duration, and average number of cigarettes smoked per day; some, such as the CONOR study also asked about age at smoking initiation. In the other surveys, age at smoking initiation was calculated both for current (age at enrollment minus duration of smoking in years) and former (age at enrollment minus years since quitting and duration of smoking in years) smokers. Current smokers were defined as those who were daily smokers, and former smokers were classified according to years since quitting smoking, or if they answered that they had smoked previously but were not smokers at the time of enrollment. We then combined the categories of current and former smokers into a single category of ever smokers. Ever smokers were further categorized according to the following factors at enrollment: age at smoking initiation in years (≤19, 20–24, ≥25), number of cigarettes smoked per day (1–9, 10–19, ≥20), smoking duration in years (1–19, 20–29, ≥30), and number of pack-years smoked (i.e., number of cigarettes smoked per day, divided by 20, multiplied by the duration of smoking in years; 0–9, 10–19, ≥20). Participants who were neither current nor former smokers were classified as never smokers and constituted the reference group throughout the present paper.

Body mass index (BMI) was calculated as weight in kilograms divided by the square of height in meters. The participants were categorized into three different groups based on the level of physical activity reported in the baseline questionnaires: sedentary (reading, watching television, and sedentary activity); moderate (walking, bicycling, or similar activities ≥4 hours a week) and heavy (heavy exercise and daily competitive sports and light sports or heavy gardening ≥4 hours). The most recent information regarding duration of education was obtained from Statistics Norway and participants were divided into three categories by duration of education in years (<10, 10–12, and ≥ 13).

### Follow-up and endpoints

We followed the participants who completed the baseline questionnaire in one of the four health surveys from 1972 until 2003 through linkage to the Cancer Registry of Norway and the Central Population Register, utilizing the unique 11-digit personal identification number to identify all cancer cases, emigrations and deaths, respectively. These national registries are both accurate and virtually complete [15,16]. The start of follow-up was set as 1 January of the year after completing the baseline questionnaire. Person-years were calculated from the start of follow-up to the date of rectal cancer diagnosis, the date of any incident cancer diagnosis (except skin basal cell carcinoma), emigration, death, or the end of follow-up, i.e., 31 December 2007, whichever occurred first. Rectal cancer was classified according to the code specified in the Seventh Revision of the International Statistical Classification of Diseases (i.e., ICD-7 code 154).

We excluded 11,476 participants who were diagnosed with any invasive cancer prior to the start of the study, and 1,009 participants who had emigrated or died before the start of follow-up. We further excluded 6,299 participants with insufficient information on smoking history. Finally, we excluded participants with missing information on BMI (n = 5,107), physical activity (n = 8,210) and education (n = 18,449), leaving 602,242 (49% men) in the analytical cohort.

### Statistical analysis

We used the *t*-test and the *χ*^2^ test to investigate differences in the distribution of selected characteristics between men and women with and without rectal cancer and between ever and never smokers. The Cox proportional hazards model was used with age as the underlying time scale to estimate multivariate-adjusted hazard ratios (HRs) with 95% confidence intervals (CIs) for the associations between different measures of smoking exposure [age at smoking initiation in years (≤19, 20–24, ≥25), number of cigarettes smoked per day (1–9, 10–19, ≥20), smoking duration in years (1–19, 20–29, ≥30) and number of pack-years smoked (0–9, 10–19, ≥20)] and rectal cancer with never smokers as the reference group. All analyses were done by gender. Entry time was defined as age at enrollment and exit time was age at diagnosis of rectal cancer, the date of any incident cancer diagnosis (except basal cell carcinoma), emigration, death, or the end of follow-up (31 December, 2007), whichever occurred first. The possible confounders included in the final models, selected a priori, were age at enrollment(continuous), level of physical activity (sedentary, moderate and heavy), BMI(continuous), all at enrollment, and duration of education in years (<10, 10–12, ≥13). Tests for linear trends were obtained by creating an ordinal exposure (including never smokers) variable with equally spaced scores and including it in the models.

We excluded 8,151 (99% men) participants who reported smoking only cigar or pipe and did a sensitivity analyses in this sub cohort. We had information on alcohol consumption for 37% (n = 221,748) of the total analytical cohort and did sensitivity analyses for the risk of rectal cancer by gender for this subcohort (49% men) with and without adjustment for alcohol consumption. Heterogeneity by gender in the effect of smoking and risk of rectal cancer was tested with the Wald *χ*^2^ test. Two-sided p -values of <0.05 were considered statistically significant. All analyses were conducted using STATA version 12.0 (Stata Corp., College Station, TX, USA).

## Results

During a mean follow up period of 14 years and 8.6 million person-years of observation, 2,176 (61% men) histologically confirmed invasive rectal cancer cases were ascertained. Mean age at rectal cancer diagnosis for men varied from 57 years in the 40 years cohort to 66 years in the CONOR and the Oslo study I and for women, it varied from 55 years in the 40 years cohort to 66 years in the CONOR study. At enrollment, 67% of men and 59% of women were ever smokers (Table 1). Compared with never smokers, both men and women ever smokers had less education, were less physically active and were leaner (all p -values <0.0001) (data not shown).

**Table 1:** Selected characteristics of the study population at enrollment, stratified by cohort, among 602,242 Norwegian men and women (1972–2003)

**Characteristics**	**Oslo study I**^**a **^**(1972–1973)**	**Norwegian counties study (1974–1987)**		**40 years cohort (1985–1999)**		**CONOR (Cohort of Norway) (1994–2003)**		**All (1974–2003)**	
	**Men**	**Men**	**Women**	**Men**	**Women**	**Men**	**Women**	**Men**	**Women**
Subjects	16,946	41,913	41,573	185,037	199,730	55,480	61,563	299,376	302,866
Person- years of follow-up	476,518	1, 058, 699	1,079, 213	2,424,435	2,595, 800	462,398	516,186	4,422, 049	4,191,200
Age at enrollment, mean, SD	45 ± 6	40 ± 7	40 ± 7	43 ± 5	43 ± 5	48 ± 14	48 ± 15	44 ± 8	44 ± 8
Age at rectal cancer diagnosis, mean, SD	66 ± 8	62 ± 8	63 ± 8	57 ± 10	55 ± 9	66 ± 11	66 ± 14	62 ± 10	59 ± 11
Year of birth, median, (Range)	1929(1925–1931)	1938(1932–1944)	1939(1932–1944)	1951(1948–1954)	1951(1948–1954)	1954(1940–1960)	1955(1941–1960)	1950(1944–1954)	1951(1946–1955)
Number of cases	286	366	281	504	426	180	133	1,336	840
Follow-up years, median, (Range)	32(24–33)	28(20–30)	30(20–31)	13(10–16)	13(10–16)	9(6–10)	9(6–10)	13(10–18)	12(9–17)
≥13 years of education^b^, (%)	24	14	12	26	22	21	21	23	20
Body mass index (BMI), mean, (kg/m^2^)	25	25	24	26	24	26	25	26	25
Level of physical activity, heavy^c^ (%)	20	31	11	35	21	38	28	34	21
Ever smokers (%)	79	74	54	66	61	62	56	67	59
Current smokers (%)	55	51	40	40	40	31	32	41	38
Former smokers (%)	24	23	14	26	21	31	24	26	21

SD standard deviation, Range interquartile range. ^a^Included only men. ^b^Not at enrollment. ^c^Heavy physical activity: Light sports or heavy gardening ≥ 4 hours per week, heavy exercise or daily competitive sports.

Table 2 shows that the multivariate adjusted HR estimate for rectal cancer was similar for current and former smokers of both genders. Ever smokers had a significantly increased risk of rectal cancer of more than 25% for both men (HR = 1.27, 95% CI = 1.11-1.45) and women (HR = 1.28, 95% CI = 1.11-1.48) compared with gender-specific never smokers. Men smoking ≥20 pack-years had a significantly increased risk of rectal cancer of 35% (HR = 1.35, 95% CI = 1.14-1.58), whereas for women it was 47% (HR = 1.47, 95% CI = 1.13-1.91) compared with gender-specific never smokers. For both men and women, we observed significant dose–response associations (including the reference category) between the four variables [age at smoking initiation in years (both p_trend_ <0.05), number of cigarettes smoked per day (both p_trend_ <0.0001), smoking duration in years (p_trend_ <0.05, <0.0001) and number of pack-years smoked (both p_trend_ <0.0001)] and rectal cancer (Table 2). The test for heterogeneity by gender was not significant between smoking status and the risk of rectal cancer (Wald *χ*2, p value; current smokers = 0.85; former smokers = 0.87; ever smokers = 1.00). These estimates did not differ materially when we excluded participants who smoked only cigars or pipes (data not shown).

**Table 2:** **Multivariate**^**a **^**adjusted hazard ratio (HR) estimates for rectal cancer with 95**% **confidence intervals (CI) among women (n = 302,866) and men (n = 299,376) according to various measures of smoking exposure at enrollment, compared with never smokers**

	**Men**				**Women**			
	**Cases n = 1,336**	**Person-years**	**HR**	**95% CI**	**Cases n = 840**	**Person-years**	**HR**	**95% CI**
Smoking status								
Never	298/98,388	1,369,691	1.00	Ref.	350/123,503	1,744,944	1.00	Ref.
Former	433/78,662	1,138,881	1.28	1.11-1.50	169/64,021	824,913	1.26	1.05-1.52
Current	605/122,326	1,913,477	1.26	1.09-1.45	321/115,342	1,621,343	1.29	1.10-1.51
*Ptrend*^b^				<0.05				<0.05
Ever	1,038/200,988	3,052,358	1.27	1.11-1.45	490/179,363	2,446,256	1.28	1.11-1.48
Ever smokers^c^								
Age at smoking initiation in years								
≥25	116/16,415	268,600	1.23	0.99-1.52	99/23,150	357,101	1.19	0.95-1.49
20-24	211/38,540	592,480	1.35	1.13-1.61	136/40,824	588,736	1.45	1.18-1.78
≤19	362/96,856	1,294,339	1.28	1.08 − 1.50	142/80,620	928,955	1.35	1.10-1.67
*Ptrend*^b^				<0.05				<0.05
Number of cigarettes smoked per day								
1-9	207/39,218	604,421	1.07	0.90-1.29	169/59,570	824,198	1.15	0.96-1.39
10-19	524/99,761	1,526,804	1.35	1.17-1.56	255/93,002	1,268,980	1.37	1.16-1.62
≥20	259/56,319	832,845	1.31	1.11-1.55	64/25,270	337,874	1.38	1.05-1.81
*Ptrend*^b^				<0.0001				<0.0001
Smoking duration in years								
1-19	326/80,190	1,250,222	1.21	1.03-1.42	220/87,999	1,263,528	1.17	0.99 − 1.40
20-29	457/97,685	1,471,526	1.29	1.11-1.50	222/81,713	1,089,772	1.37	1.15-1.64
≥30	232/21,144	299,518	1.31	1.09-1.59	48/7,918	76,398	1.54	1.11-2.12
*Ptrend*^b^				<0.05				<0.0001
Number of pack-years smoked^d^								
0-9	298/68,003	943,796	1.17	0.99-1.37	241/88,884	1,270,193	1.21	1.02-1.42
10-19	385/74,235	1,014,305	1.33	1.14-1.54	178/64,544	862,029	1.38	1.14-1.66
≥20	302/52,392	647,100	1.35	1.14-1.58	69/23,263	288,147	1.47	1.13-1.91
*Ptrend*^b^				<0.0001				<0.0001

^a^Adjusted for age, body mass index, level of physical activity all at enrollment and duration of education. ^b^Never smokers included in the model. ^c^Total numbers of ever smokers do not equal the total in different smoking exposures due to missing values in different smoking exposures groups. ^d^Pack-years were calculated as numbers of cigarettes smoked per day, divided by 20 and multiplied by the number of years smoked.

In the sensitivity analyses for men with information on alcohol consumption most of whom were enrolled after 1995, the risk estimate of rectal cancer incidence was 13% (HR = 1.13, 95% CI = 0.83-1.55) with adjustment for alcohol consumption and 12% (HR = 1.12, 95% CI = 0.82-1.54) without adjustment for alcohol consumption among ever compared with never smokers. The same analyses among women rendered a risk estimate of 37% (HR = 1.37, 95% CI = 0.99-1.92) with adjustment for alcohol consumption and 39% (HR = 1.39, 95% CI = 1.00-1.94) without adjustment for alcohol consumption.

Table 3 shows that among men, ever smokers had a significantly increased risk of rectal cancer compared with gender-specific never smokers for all three levels of BMI (<25, 25–29, ≥ 30), duration of education in years (<10, 10–12, ≥13) and level of physical activity (sedentary, moderate and heavy). For women, the corresponding figure was significantly increased for eight of the nine displayed categories (Table 3).

**Table 3:** **Age and multivariate**^**a **^**adjusted HR estimates for rectal cancer with 95**% **CI among 602,242 Norwegian men and women ever smokers according to selected covariates and never smokers as reference group**

	**Men**		**Women**	
**Ever smokers**	**Cases n = 1038**	**Multivariate adjusted**^**a **^**HR (95% CI)**	**Cases n = 490**	**Multivariate adjusted**^**a **^**HR (95% CI)**
Body mass index (kg/m^2^)				
<25	484	1.17(1.01-1.36)	296	1.18(1.01-1.39)
25-29	466	1.33(1.15-1.54)	145	1.39(1.15-1.70)
≥30	88	1.53(1.20-1.95)	49	1.39(1-15-1.70)
Duration of education (years)^b^				
<10	356	1.20(1.02-1.40)	185	1.22(1.02-1.47)
10-12	497	1.26(1.09-1.45)	248	1.31(1.11-1.55)
≥13	185	1.41(1.17-1.70)	57	1.28(0.96-1.70)
Level of physical activity^c^				
Sedentary	241	1.36(1.15-1.62)	125	1.30(1.06-1.60)
Moderate	550	1.27(1.10-1.46)	292	1.24(1.05-1.46)
Heavy	247	1.22(1.03-1.45)	73	1.39(1.07-1.79)

^a^Adjusted for age, body mass index, physical activity all at enrollment and duration of education. ^b^Not at enrollment. ^c^Level of physical activity; sedentary (reading, watching television, and sedentary activity), moderate (walking, bicycling, or similar activities ≥4 hours per week), and heavy (light sports or heavy gardening ≥4 hours per week, heavy exercise or daily competitive sports).

## Discussion

Our study shows that ever smokers had a significantly increased risk of rectal cancer, and that this risk is similar for men and women. A possible causal interpretation of our results is supported by the presence of a consistent dose–response association between the various measures of smoking exposure (i.e., age at smoking initiation in years, number of cigarettes smoked per day, smoking duration in years and number of pack-years smoked) and the risk of rectal cancer for both genders. Men and women ever smokers also had an increased risk of rectal cancer within the different categories of possible confounding variables, such as BMI, duration of education and level of physical activity.

To our knowledge, this prospective analysis of smoking and the risk of rectal cancer includes the largest number of rectal cancer cases investigated to date. It is also the first to compare this association in detail by gender. In the present report, the association between cigarette smoking and rectal cancer was similar for men and women. Previously, we reported from the same cohort that smoking increased the risk of colon cancer to a greater extent for women than men [2]. Our present findings of no difference between the gender in the smoking related increased risk of rectal cancer are in accordance with three [17-19] smaller Japanese cohort studies including 200 cases of rectal cancer [19] or less [17,18]. The European Prospective Investigation into Cancer and Nutrition (EPIC) cohort with 950 incident rectal cases among almost half a million men and women from ten European countries [20], and the Singapore Chinese Health Study with 329 rectal cancer cases are the largest cohort studies including both genders before ours examining the association between smoking and the risk of rectal cancer [21]. The former study found a non-significant increase in rectal cancer among ever smokers [20] whereas the latter study found a significantly increased risk [21]. Neither of these two studies reported on the smoking-related risk of rectal cancer by gender. Four other cohort studies included either only women [22-24], or only men [25]. The studies, from Canada [24] and the United States [23] showed slightly higher risk estimates than ours, whereas the studies from Norway [22], and Korea [25], had lower risk estimates. The association between smoking and rectal cancer achieved statistical significance only among current smokers in the United States [23] and among former smokers in the Canadian study [24]. In our study, for both genders, former, current and ever smokers all had a significantly increased risk of rectal cancer.

Two meta-analyses, one including 36 prospective cohort studies reported a non-significant almost 20% increased risk of rectal cancer for both former and current smokers [26] while the other comprised 106 observational studies and reported a significantly increased risk of rectal cancer of 25% among ever smokers [27]. Neither of these meta-analyses reported gender-specific risk estimates.

In studies reporting risk estimates by cancer site, the association between smoking and rectal cancer has generally been stronger than that with colon cancer among both men and women. Similarly, stronger relative risk among ever smokers for proximal compared to distal colon cancer has been documented [1]. In our previous study [2], we found that the smoking-related risk of colon cancer was more pronounced in the proximal part of colon for women, but not for men. For the distal part of colon, we could not demonstrate a difference by gender. These results, as well as those reported in the IARC monograph, are in accordance with the findings of the present study.

Colorectal cancer is considered a complex collection of diseases with different etiologies [28]. Smoking causes irreversible genetic damage in the colorectal mucosa due to its carcinogenic effects, which lead to cancerous changes. In 1996 Giovannucci et al. [29] hypothesized that smoking is an initiator of colorectal carcinogenesis, but that the increased risk only emerges 30 to 40 years after smoking initiation. In an updated review study from 2001, Giovannucci [30] reiterated his stand on this issue, stating that the induction period could be from 35 to 40 years. The notion that cigarette smoking is considered an initiator rather than a promoter of rectal cancer was also supported in the study by Terry et al. [24]. Our results showed a significantly increased risk of rectal cancer for smokers who had smoked for <20 years at enrollment for men and <30 years for women. When we add the median follow-up time of 13 years for men and 12 years for women, our results showed an induction period that is in accordance with the above suggestions.

Our study has several major strengths. It is based on a large prospective cohort population from Norway comprising both men and women, with a long and virtually complete follow-up. The long follow-up period resulted in a large number of cases, and gave us more stable risk estimates and results that are less prone to chance. We were able to stratify all the analyses according to different measures of smoking exposure and were able to conduct all analyses separately by gender. Also, smoking histories were obtained at enrollment and, therefore, are not subject to recall bias. We have a high proportion of men and women ever smokers. In addition, we focused our analyses on the comparison between ever versus never smokers. Thus, it is only never smokers that could have possibly changed their smoking status during follow-up. As very few Norwegians start to smoke after the age of 30 and the mean age at enrollment for our study is more than 40 years, we are confident that possible changes in smoking status among never smokers during follow-up did not influence our risk estimates. We had information on, and were able to control for, established risk factors for rectal cancer, many of which varied according to smoking status. Rectal cancer screening was not in place in Norway during our study period, thus reducing detection bias. Also, two previous reports confirmed the internal validity of the association between smoking exposure and the risk of breast [14] and colon cancer [2].

Our study has also several limitations. We lacked information on family history of rectal cancer and on dietary factors, such as alcohol and red meat consumption, which are established risk factors for rectal cancer. Increased consumption of alcohol and red meat are factors that may partly explain the steep increase in rectal cancer incidence for both genders. Alcohol consumption is higher among men than women in Norway [31]. Thus, the lack of adjustment for alcohol consumption in our main analyses is likely to have inflated the estimates among men more than women, thereby, biasing a potential gender difference. However, in the subcohort analyses, the risk estimates were similar for men ever smokers with and without adjustment for alcohol consumption. This was also the case for women. This indicates that our results may be noteworthy in spite of the lack of data on alcohol consumption for the majority of the subjects in the main analyses. Rectal cancer has a long induction period [30] and the interpretation of our sensitivity analyses should be done with caution, as they included fewer cases and younger participants with less follow-up time compared to the main cohort. If Norwegian men consumed more red meat than women, this would bias a potential gender difference in the same direction as alcohol consumption. However, we cannot rule out that alcohol and red meat consumption may have stronger effects in women than men.

Similarly, information on the use of COX inhibitors, such as aspirin, which has preventive effects on rectal cancer development [32] was not available. The lack of molecular data is another limitation. We also lacked detailed information on occasional and passive smoking. From 1976 to 2006, which is during our follow-up period, around 10% of the Norwegian population reported smoking occasionally [33]. We believe that some occasional smokers may have been excluded due to insufficient smoking information, whereas others may have been included in the reference group, together with women exposed to passive smoking, which would have attenuated the associations between smoking and rectal cancer. As current smokers have an increased risk of dying from any major cause during follow-up and rectal cancer is assumed to take many years, competing causes of death may decrease the impact of smoking more among current than former smokers, and make the association with rectal cancer more similar for current and former smokers. There may be some residual confounding due to these and other unknown risk factors. Nevertheless, the dose–response association we observed is suggestive of a causal association between smoking and rectal cancer for both men and women.

## Conclusions

Smoking increases the risk of rectal cancer to the same extent in women as in men.

## Abbreviations

BMI: Body mass index; CI: Confidence interval; CONOR: Cohort of Norway; EPIC: European Prospective Investigation into Cancer and Nutrition; IARC: International Agency for Research on Cancer; ICD: International classification of diseases; HR: Hazard ratio; SD: Standard deviation.

## Competing interests

The authors declare that they have no competing interests.

## Authors’ contributions

RP carried out the statistical analysis and drafted the manuscript. ITG, EW and EB contributed to the planning of the manuscript, statistical analysis, interpretation of the data and critical revision of the manuscript. AT contributed with statistical analysis interpretation of data and critical revision of the manuscript. LM contributed with interpretation of the data and critical revision of the manuscript. All authors read and approved the final version of the manuscript.

## Pre-publication history

The pre-publication history for this paper can be accessed here:

http://www.biomedcentral.com/1471-2407/14/321/prepub
